# Genomic Analysis Reveals a Potential Role for Cell Cycle Perturbation in HCV-Mediated Apoptosis of Cultured Hepatocytes

**DOI:** 10.1371/journal.ppat.1000269

**Published:** 2009-01-16

**Authors:** Kathie-Anne Walters, Andrew J. Syder, Sharon L. Lederer, Deborah L. Diamond, Bryan Paeper, Charles M. Rice, Michael G. Katze

**Affiliations:** 1 Department of Microbiology, School of Medicine, University of Washington, Seattle, Washington, United States of America; 2 Laboratory of Virology and Infectious Disease, Center for the Study of Hepatitis C, Rockefeller University, New York, New York, United States of America; Harvard Medical School, United States of America

## Abstract

The mechanisms of liver injury associated with chronic HCV infection, as well as the individual roles of both viral and host factors, are not clearly defined. However, it is becoming increasingly clear that direct cytopathic effects, in addition to immune-mediated processes, play an important role in liver injury. Gene expression profiling during multiple time-points of acute HCV infection of cultured Huh-7.5 cells was performed to gain insight into the cellular mechanism of HCV-associated cytopathic effect. Maximal induction of cell-death–related genes and appearance of activated caspase-3 in HCV-infected cells coincided with peak viral replication, suggesting a link between viral load and apoptosis. Gene ontology analysis revealed that many of the cell-death genes function to induce apoptosis in response to cell cycle arrest. Labeling of dividing cells in culture followed by flow cytometry also demonstrated the presence of significantly fewer cells in S-phase in HCV-infected relative to mock cultures, suggesting HCV infection is associated with delayed cell cycle progression. Regulation of numerous genes involved in anti-oxidative stress response and TGF-β1 signaling suggest these as possible causes of delayed cell cycle progression. Significantly, a subset of cell-death genes regulated during *in vitro* HCV infection was similarly regulated specifically in liver tissue from a cohort of HCV-infected liver transplant patients with rapidly progressive fibrosis. Collectively, these data suggest that HCV mediates direct cytopathic effects through deregulation of the cell cycle and that this process may contribute to liver disease progression. This *in vitro* system could be utilized to further define the cellular mechanism of this perturbation.

## Author Summary

Chronic HCV infection is associated with progressive liver injury and subsequent development of fibrosis/cirrhosis. The cellular mechanisms by which HCV replication, and subsequent virus–host interactions, may mediate liver injury are unclear. Microarray experiments were performed to characterize the host transcriptional response to HCV infection of cultured hepatocytes in an attempt to gain insight into the mechanism of HCV-associated cell death. Analysis of the gene expression data revealed that many differentially regulated genes function to induce apoptosis in response to cell cycle arrest, possibly in response to DNA damage and oxidative stress. Labeling of dividing cells in culture followed by flow cytometry also demonstrated the presence of significantly fewer cells in S-phase in HCV-infected cultures relative to mock cultures, suggesting HCV infection is associated with delayed cell cycle progression. Finally, many of the cell-death–related genes whose expression changes in response to HCV infection of cultured hepatocytes were also differentially regulated in liver tissue from HCV-infected patients with histological evidence of fibrosis. In summary, HCV may mediate direct cytopathic effects through perturbation of the cell cycle which potentially contributes to liver disease progression.

## Introduction

Hepatitis C virus (HCV), a member of the Flaviviridae family, is a blood-borne pathogen which currently infects approximately 170 million people worldwide. Exposure to HCV typically results in a persistent infection and approximately 30% of chronically infected patients will develop progressive liver disease including fibrosis, cirrhosis and hepatocellular carcinoma (HCC) [1]. The majority of pathology associated with chronic infection is believed to occur via a HCV-specific cell-mediated immune response [2]. However, in light of this, it is somewhat perplexing that liver disease progression is accelerated in immuno-compromised individuals. Specifically, HCV/HIV-coinfected patients and liver transplant patients receiving immuno-suppressive drugs tend to develop fibrosis/cirrhosis at a much faster rate than immuno-competent individuals [3]. A recent study found that HCV-specific CD8 T cells were actually associated with areas of low hepatocellular apoptosis and weak fibrosis. It is thought that these cells are protective of liver damage through production of IL-10 [4]. Furthermore, characterization of the host response to HCV infection in the SCID-Alb/uPA mouse model demonstrated histological evidence of hepatocyte apoptosis in a manner similar to that observed during acute HCV infection in patients [5]. HCV infection in this model was associated with perturbations in cellular pathways, including lipid metabolism and oxidative stress, which have the potential to be cytopathic. The inability of these animals to generate a virus-specific immune response raises the intriguing possibility that HCV replication is capable of directly mediating hepatocyte apoptosis.

It is now thought that both direct cytopathic effects and immune-mediated processes likely play a role in HCV-associated liver injury [3]. The cellular mechanisms by which HCV replication, and subsequent virus-host interactions, may mediate liver injury are unclear. Progress in this area has been hindered by the lack of appropriate model systems in which to investigate the role of viral factors in liver disease progression. Currently, studies focused on defining the mechanisms of HCV-associated liver injury are primarily restricted to limited analysis of patient samples, including liver biopsy tissue. While such studies have provided significant insight into the role of steatosis, oxidative stress and death-receptor signaling in liver disease, there are obvious limitations with respect to conducting more mechanistic studies, in particular the role of viral proteins.

There is a wealth of literature describing experiments in which one more HCV proteins are over expressed in cultured hepatocytes. The results indicate a wide range of, and often conflicting, effects of viral protein expression on cellular functions, including apoptosis (Reviewed in Fischer et al, 2007). Core protein, in particular, has been reported to have both pro- and anti-apoptotic effects on death-ligand mediated hepatocyte apoptosis, including TNF-α, CD95Ligand and TRAIL-induced apoptosis, via a variety of mechanisms. The HCV envelope protein E2 has been found to both inhibit TRAIL-induced apoptosis and also to induce mitochondria-related/caspase-dependent apoptosis in the same hepatoma cell line. Perturbations of apoptotic pathways have also been demonstrated with the non-structural proteins. The NS3 protease inhibits pro-apoptotic RIG-I signaling via cleavage of the adaptor protein Cardif and also induces apoptosis of hepatocytes via caspase-8. NS5A has been found to inhibit apoptosis through multiple mechanisms, including sequestering of p53, activation of NFkB, increased expression of bcl-XL and p21 as well as activation of the P13-kinase-AKt/PKB survival pathway. While intriguing, many of these studies involve the expression of a single HCV protein, often at very high levels which do not accurately represent those seen in naturally infected livers. These experiments also fail to study the impact of potentially crucial interactions between the different HCV proteins.

A significant breakthrough in HCV research was achieved by the discovery of a specific HCV strain that efficiently infects and replicates in the cultured hepatoma cell line Huh-7.5 [6],[7],[8],[9]. This strain, termed JFH-1, was isolated from a Japanese patient who suffered fulminant hepatitis following exposure to the virus [6],[7]. Subsequent inoculation of clonal JFH-1 into chimpanzees and SCID-Alb/uPA mice resulted in productive infections in the absence of fulminant hepatitis, suggesting that the host response to infection played a key role in the severe form of hepatitis observed in this patient [8]. This model system provides the opportunity to study the impact of viral protein expression and replication on host cell function during a productive HCV infection and to potentially investigate the role of viral factors in liver injury. In the current study, microarray experiments were performed to characterize the host transcriptional response to HCV infection in an attempt to gain insight into the mechanism of HCV-associated cell death. Both the presence of activated caspase-3 and induction of cell death-related genes indicated that HCV infection was associated with a direct cytopathic effect. Gene ontology analysis suggests a role of cell cycle perturbation, possibly in response to oxidative stress and/or TGF-β1 signaling, in HCV-mediated apoptosis.

## Results

### HCV-J6/JFH Infection of Huh-7.5 Cells

**Figure 1:**
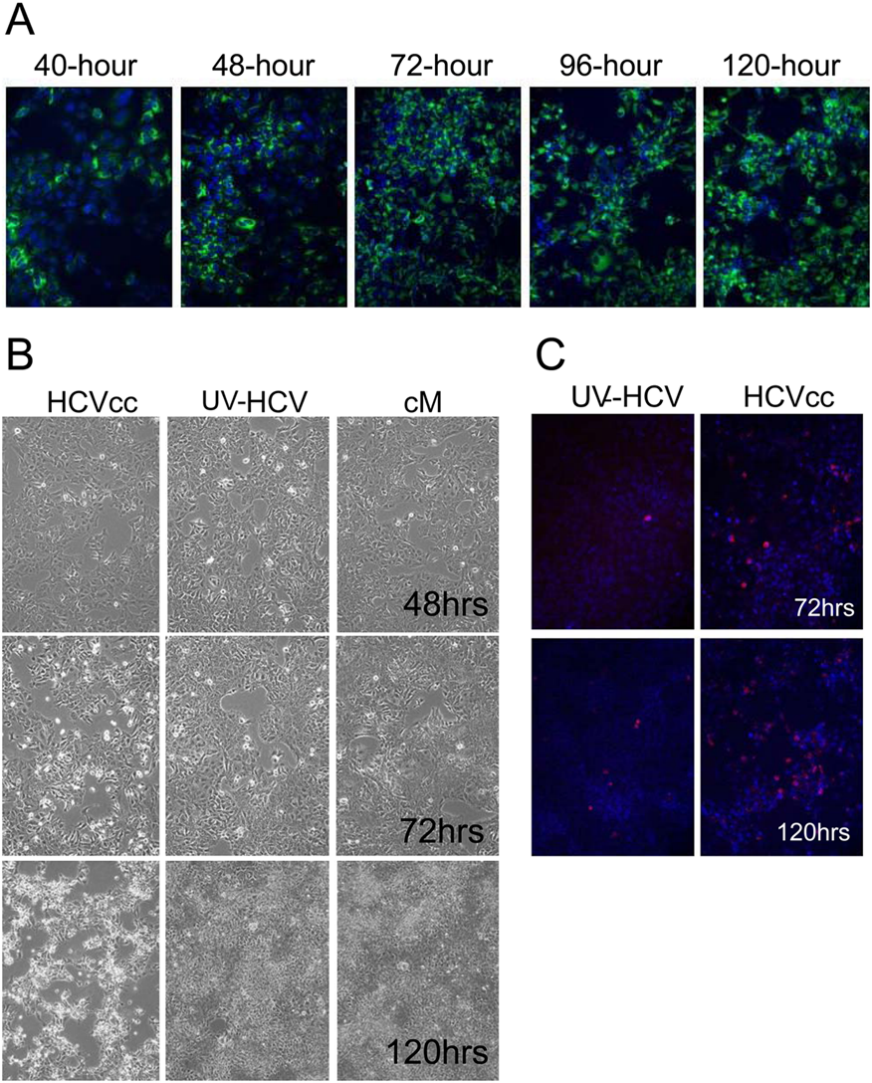
Characterization of HCV-JFH infection of Huh-7.5 cells. (A) Expression of HCV NS5A in Huh-7.5 cells. HCV (+) cells are detected using an anti-NS5A antibody (green) while all cell nuclei are shown using Hoechst dye (blue). (B) Cytopathic effect observed during later time-points of HCV infection in Huh-7.5 cells. (C) Presence of activated caspase-3 (red) as shown by immuno-fluorescence staining of HCV-infected Huh-7.5 cells. Nuclei are shown using Hoechst dye (blue).

To study the host response to infection during the early phase of acute infection, Huh-7.5 cells were infected at a relatively high MOI (1–2 virions/cell) with HCV genotype 2a chimeric virus, J6/JFH (HCVcc) [7]. The virus used to infect cells was a pool of cell culture adapted virions generated by multiple passages over naïve Huh 7.5 cells (see Materials and Methods). For infection controls, cells were inoculated with either UV-inactivated HCVcc (to distinguish effects due to virus binding and virus replication) or conditioned media (mock). Conditioned media was used for the mock as it has been shown that factors present in the media of cultured cells can induce transcriptional changes (Walters, unpublished data). Cells were incubated with HCVcc for approximately 8 hrs, after which the cells were washed and fresh media added. Following infection, HCV (+) cells were visualized using an anti-NS5A antibody. As shown in Figure 1A, the majority of the cells expressed viral antigen by 48 hrs post-infection and continue to do so for the remainder of the study. No HCV RNA or viral protein expression was detected in cells exposed to UV-inactivated HCVcc (data not shown). Samples were harvested at 24, 48, 72, 96, and 120 hours post-infection and cellular RNA isolated for measuring intracellular HCV RNA levels and for global gene expression profiling.

### Acute HCV Infection Induces Apoptosis of Cultured Hepatoma Cells

**Figure 2:**
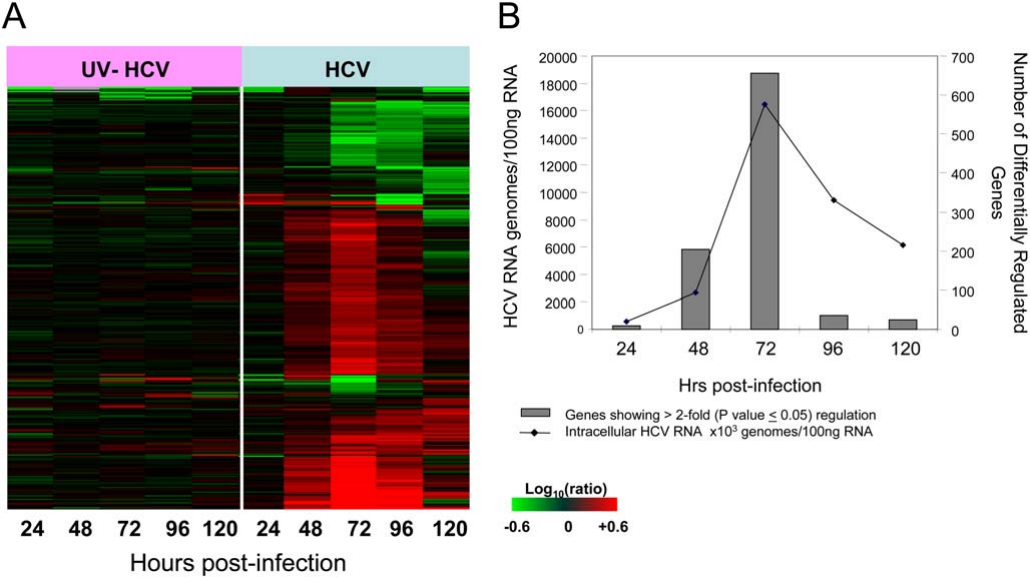
Host transcriptional response to acute HCV infection of Huh-7.5 cells. (A) Expression profiles of 860 sequences that are regulated (2-fold, P value<0.05) in at least 1 experiment. Each column represents gene expression data from an individual experiment comparing either UV-inactivated HCVcc or HCVcc-treated cells relative to time-matched, mock-treated Huh-7.5 cells. Genes shown in red were up-regulated, genes shown in green down-regulated, and genes in black indicate no change in expression in HCV-infected cells relative to uninfected cells. (B) Association between intracellular HCV RNA levels and effect on host gene expression in HCV-infected Huh-7.5 cells. Intracellular HCV RNA levels were determined by quantitative RT-PCR as described in Materials and Methods. Total number of genes showing differential regulation (2-fold, P value<0.05) in HCV-infected cells relative to uninfected cells is shown in black line while the HCV viral load are shown using grey bars. Cells exposed to UV-inactivated HCVcc show negligible virus and gene expression changes and so are not shown.

Similar to what has been reported previously, a cytopathic effect was observed in the cultures of cells infected with HCVcc starting around 72 hrs post-infection (Figure 1B). This effect was not observed in the cells exposed to either UV-inactivated HCVcc or conditioned media, indicating that it is induced by active viral replication. The cytopathic effect became more prominent at 96 hrs and appeared to include the majority of the cells by 120 hrs post-infection (Figure 1B). Immuno-histochemistry specific for cleaved caspase-3 demonstrated activation of a terminal pathway involved in apoptosis that appears to cause the cytopathic effect in culture. As shown in Figure 1C, cleaved caspase-3 was present in cells infected with HCVcc beginning around 72 hrs, suggesting that the mechanism of cell death was apoptosis. It was not observed in cells exposed to either conditioned media (data not shown) or UV-inactivated virus. The initial presence of activated caspase-3 also coincided with peak levels of intracellular viral RNA (Figure 2B), suggesting a causative link between the level of HCV replication and cell death.

### Global Transcriptional Response to Acute HCV Infection of Cultured Hepatoma Cells

Microarray experiments were performed to characterize the host transcriptional response to HCV infection in an attempt to gain insight into the mechanism of HCV-associated cell death. For these experiments, mRNA samples isolated from cells exposed to either HCVcc or UV-inactivated HCVcc were compared to mRNA isolated from time-matched mock-treated cells. Figure 2A shows the global gene expression profiles of cells infected with UV-inactivated HCVcc and HCVcc at 24, 48, 72, 96, and 120 hrs post-infection. Similar to what was observed in previous genomic studies using the chimpanzee and SCID-Alb-uPA mouse models, the overall effect of HCV infection on cellular gene expression was subtle in the early phases of infection, with less than 50 differentially regulated genes at 24 hrs post-infection. Overall, 860 genes showed a 2-fold or higher change in expression (P value≤0.05) in at least one experiment (Figure 2A). The primary sequence name and fold-change of these genes are shown in Table S1. In contrast, cells exposed to the UV-inactivated virus showed very little, if any, regulation of genes (2-fold change, P value≤0.05) throughout the time-course, indicating that the process of virus attachment and entry into cells does not significantly impact host cell gene expression. A similar lack of differential regulation was observed in global transcriptional profiling of cells containing the HCV full-length replicon (data not shown). As shown in Figure 2B, there was a clear association between intracellular HCV RNA levels and number of differentially expressed genes, with the maximum regulation of cellular genes coinciding with peak intracellular HCV RNA levels (72 hrs post-infection). The reason for the decreasing HCV RNA levels following 72 hrs is unclear but may be related to a decrease in the number of cells capable of producing high levels of virus. It is interesting to note that the majority of transcriptional changes involve increased expression of host genes. Few differentially expressed genes showed decreased expression in the HCV-infected relative to the mock-infected cultures (Figure 2A), although the significance of this finding is unclear.

### Potential Role of Cell Cycle Perturbation in HCV-Mediated Apoptosis of Huh-7.5 Cells

Gene ontology analysis was used to identify the cellular processes represented by the changes in steady-state abundance of transcripts associated with HCV infection. Notably, many of the differentially expressed genes belong to functional categories of cell death, cell cycle and cell growth/proliferation. Indeed, cell death genes comprised approximately half of all annotated differentially regulated genes at each time-point, with the exception of 24 hrs post-infection which showed negligible regulation of cellular genes. Figure S1 demonstrates the expression profiles of 118 genes associated with cell death in HCV-infected cells. Differential expression occurred beginning at 48 hrs post-infection at which time there was no significant visual evidence of cytopathic effect or apoptosis. Maximum differential expression of cell death genes occurred at 72 hrs post-infection whereas the level of apoptosis, as measured by caspase-3 cleavage, continued to increase until 120 hrs post-infection.

**Figure 3:**
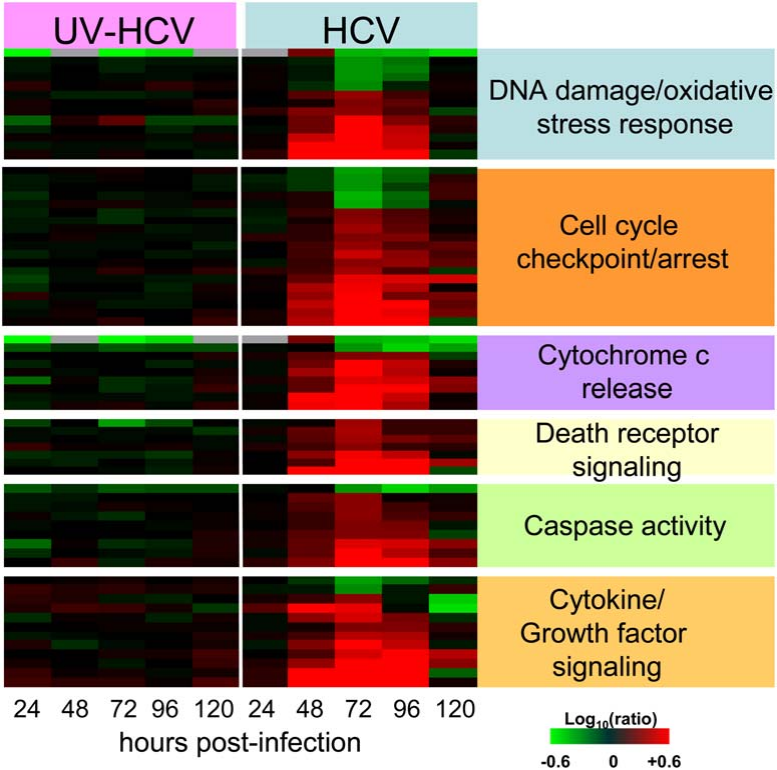
Gene ontology analysis and expression of a subset of cell-death–related genes regulated during HCV infection. Gene annotation was performed using Ingenuity Pathway Analysis and Entrez gene. Heatmap shows expression profiles of sequences that are regulated (2-fold, P value<0.05) in at least 1 experiment. Each column represents gene expression data from an individual experiment comparing either UV-inactivated HCVcc or HCVcc-treated cells relative to time-matched, mock-treated Huh-7.5 cells. Genes shown in red were up-regulated, genes shown in green down-regulated, and genes in black indicate no change in expression in HCVcc-infected cells relative to uninfected cells.

Both the differential expression of cell death-related genes and detection of cleaved caspase-3 in HCV-infected cells indicated that the observed cytopathic effect is apoptosis. Ingenuity Pathway Analysis identified a large number of cell death-related genes that function in cell cycle checkpoint/arrest, suggesting a potential role of cell cycle perturbation in apoptosis of infected cells (Figure 3). Consistent with this, a significant number of genes were identified that are associated with the DNA damage/oxidative stress response, many of which belong to the NRF2-mediated oxidative stress response pathway. This suggests that HCV replication is associated with generation of reactive oxygen species (ROS). Interestingly, the expression of two members of this pathway (CAT and EPHX1) was decreased, suggesting the ability of the cells to deal with excess ROS may be impaired. Apoptosis associated with cell cycle arrest is thought to be mediated through the mitochondria and p53 pathway [10]. In support of this, there was regulation of genes that have been linked to cytochrome c release from mitochondria (BBC3, BIK, BMF, PMAIP1, GSN, HRK). Many of these genes, along with others (DAPK3, CASP4, RIPK2), are also known to specifically regulate caspase activation. Interestingly, Ingenuity Pathway Analysis revealed that p53 signaling was significantly effected during HCV infection and this could provide the important link between oxidative stress/DNA damage, cell cycle arrest and apoptosis. Indeed, many of the differentially expressed genes associated with HCV infection that are involved in cell cycle arrest (TP53INP1, GADD45A, GADD45B, KLF6, UHRF1) and DNA damage/oxidative stress response (PMAIP1, ATF3, BBC3, FOXO3A, NOXA, CAT, UHRF1) regulate apoptosis via interaction with p53.

### HCV Infection Is Associated with Delayed Cell Cycle Progression

**Figure 4:**
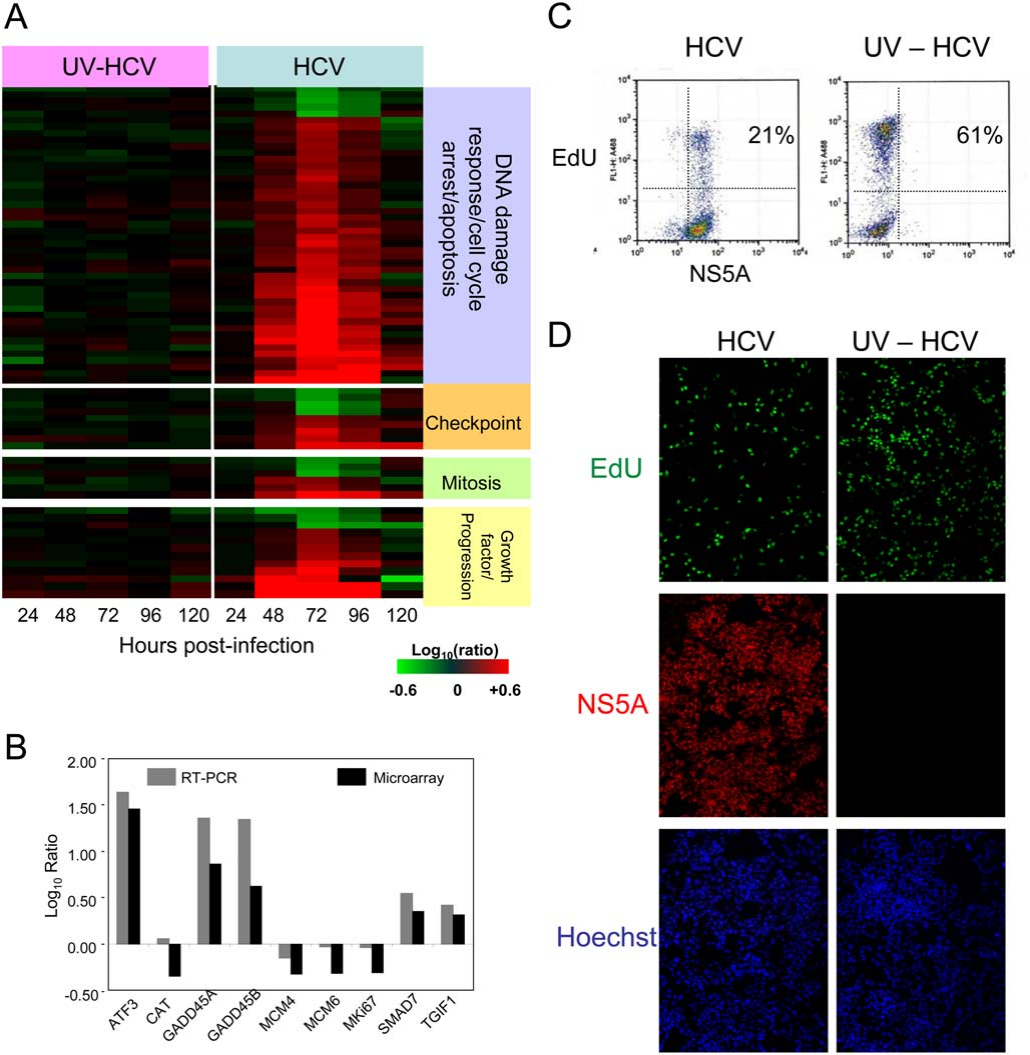
Cell cycle analysis during HCV infection. (A) Gene annotation was performed using Ingenuity Pathway Analysis and Entrez gene. Expression profiles of sequences that are regulated (2-fold, P value<0.05) in at least 1 experiment. Each column represents gene expression data from an individual experiment comparing either UV-inactivated HCV or HCV-treated cells relative to time-matched, mock-treated Huh-7.5 cells. Genes shown in red were up-regulated, genes shown in green down-regulated, and genes in black indicate no change in expression in HCV-infected cells relative to uninfected cells. (B) RT-PCR validation of the expression array data. Data are shown as log_10_ ratio and reflect the difference in expression between HCV-infected (72 hours post-infection) and mock Huh-7.5 cells. (C,D) Analysis of proliferating cells at 72 hours post-infection with HCV or UV-inactivated virus (UV-HCV). Following a 3-hour pulse with the nucleoside analog EdU, cells were enumerated by flow cytometry (C) or visualized as adherent cells using immunofluorescence (D).

The functional categories cell cycle and cell growth/proliferation were also significantly enriched among genes showing differential expression during HCV infection. However, the majority of the differentially expressed genes associated with cell cycle regulation were involved with cell cycle checkpoint/arrest and subsequent induction of apoptosis, rather than actual progression through the cell cycle (Figure 4A). This likely explains the significant overlap between cell cycle genes and those associated with cell death as described above. Many of the genes associated with checkpoint/arrest involved the G1/S phase transition, suggesting that this checkpoint is the main area of cell cycle regulation by HCV replication. Similar to what was observed with apoptosis-associated genes, many cell cycle genes function to induce cell arrest in response to DNA damage and cellular stress. Genes previously identified as transcriptional targets of p53-induced growth arrest and apoptosis (BBC3, PMAIP1, AURKB, MKi67, RRM2, MCM4, and MCM6) were also differentially regulated in HCV-infected cells. Again this suggests that perturbations in the p53 signaling pathway play a key role in perturbation of cell cycle and induction of apoptosis during HCV infection [11],[12]. Increased expression of genes encoding proteins which function to either decrease p53 levels or serve as a protective effect against p53-dependent apoptosis (TYMS, JUND, and UBD) suggests the cell is attempting to counteract the activation of the p53 signaling pathway. Alternatively, the cell may be trying to undergo apoptosis and the virus is trying to counteract the process. A much smaller set of genes associated with mitosis and cell cycle progression (positive regulators of cell proliferation) were also differentially regulated. Interestingly, the expression of MK167, Mcm4 and Mcm6, common markers of cell proliferation, are decreased during HCV infection. This is consistent with the observation that proliferation of HCV-infected Huh-7.5 cells was slower than naïve cells and provides further support that HCV-infection delays cell cycle progression (data not shown). Quantitative PCR analysis of a number of the genes shown in Figure 4A demonstrated a good correlation with the gene expression data from the microarrays (Figure 4B), although the ratios calculated from RT-PCR generally exceeded those obtained using microarrays.

Flow cytometry analysis was performed to determine if HCV infection was associated with alterations in the cell cycle. Specifically, the number of cells progressing through S-phase of the cell cycle was determined by pulse-labeling the cells with the nucleoside EdU (5-ethynyl-2′-deoxyuridine), followed by a copper-catalyzed covalent reaction to fluorescently detect the DNA-incorporated nucleoside analog (see Materials and Methods). At 72-hours post-infection, approximately 21% of the HCV-infected population showed evidence of EdU incorporation/DNA synthesis, compared to 61% of the UV-inactivated control cells (Figure 4C). This significant reduction of labeled cells in the HCV-infected population suggests reduced cellular proliferation, or a block in cell cycle progression prior to S-phase, due to the presence of the virus. Comparable results were obtained when performing the analysis by immuno-fluorescence on fixed/attached cells (Figure 4D). Reduced cell proliferation due to HCV could also be seen at earlier time-points (24 and 48 hours post-infection), but the difference was not as dramatic, although it was progressive (data not shown).

### Cytokines Induced during Acute *In Vitro* HCV Infection Are Associated with Disease Progression in Patients

**Figure 5:**
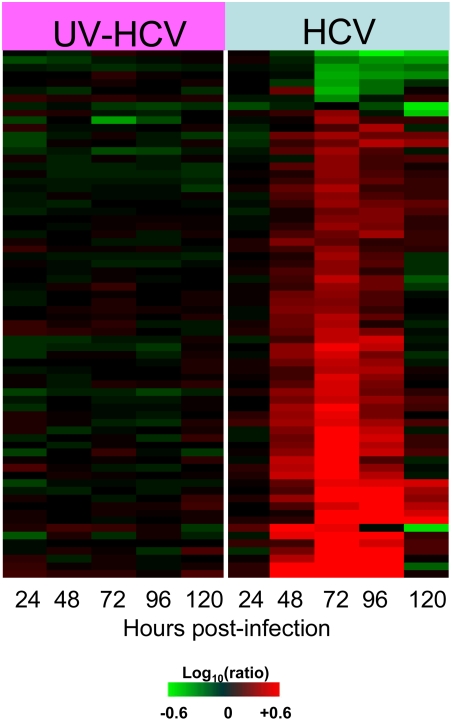
Expression profiles of cytokine/growth factor signaling-related genes in HCV-infected Huh-7.5 cells. Heatmap depicts 70 genes regulated (2-fold, p value<0.05) in at least 1 experiment. Each column represents gene expression data from an individual experiment comparing either UV-inactivated HCV or HCV –treated cells relative to time-matched mock-treated Huh-7.5 cells. Genes shown in red were up-regulated, genes shown in green down-regulated and genes in black indicate no change in expression in HCV-infected cells relative to uninfected cells.

HCV infection was also associated with differential expression of genes associated with cytokine/growth factor signaling, with the highest induction again at 72 hours post-infection (Figure 5). These genes included pro-inflammatory cytokines which are chemotactic for specific immune cells (e.g. CCL4-macrophages, CXCL1-neutrophils, IL8/CXCL2/CXCL3-PMNs, CX3CL1-macrophages, NK, lymphocytes, and CCL20-dendritic/lymphocytes). Of particular interest, some of the cytokines induced by HCV infection *in vitro* (including CCL4, CXCL1, IL32, TGFβI, TNFRSF12A, SOCS3 and TNFSF14) were identified by ANOVA analysis as being significantly (P value<0.01) associated with fibrosis progression in these transplant patients (data not shown). The fact that they are induced in HCV-infected Huh-7.5 cells suggests that hepatocytes themselves are an important source of these cytokines in an HCV-infected liver. The induction of SOCS2 and SOCS3, negative regulators of cytokine signaling, may indicate that the hepatocytes are actually trying to attenuate the expression of cytokines, possibly because they are negatively impacting cell viability. Interestingly, despite the lack of both TLR3 and a functional RIG-I in Huh-7.5 cells, induction of known interferon stimulated genes (ISGs), including ISG15 and ISG20, was also observed. Comparison of the gene expression profiles of HCV-infected and IFN-treated Huh-7.5 cells revealed significant overlap in differentially regulated genes, suggesting that HCV infection is associated with activation of Type 1 IFN signaling (data not shown). As the cultures were nearly 100% infected, the expression of these genes is likely coming from HCV-infected hepatocytes.

**Figure 6:**
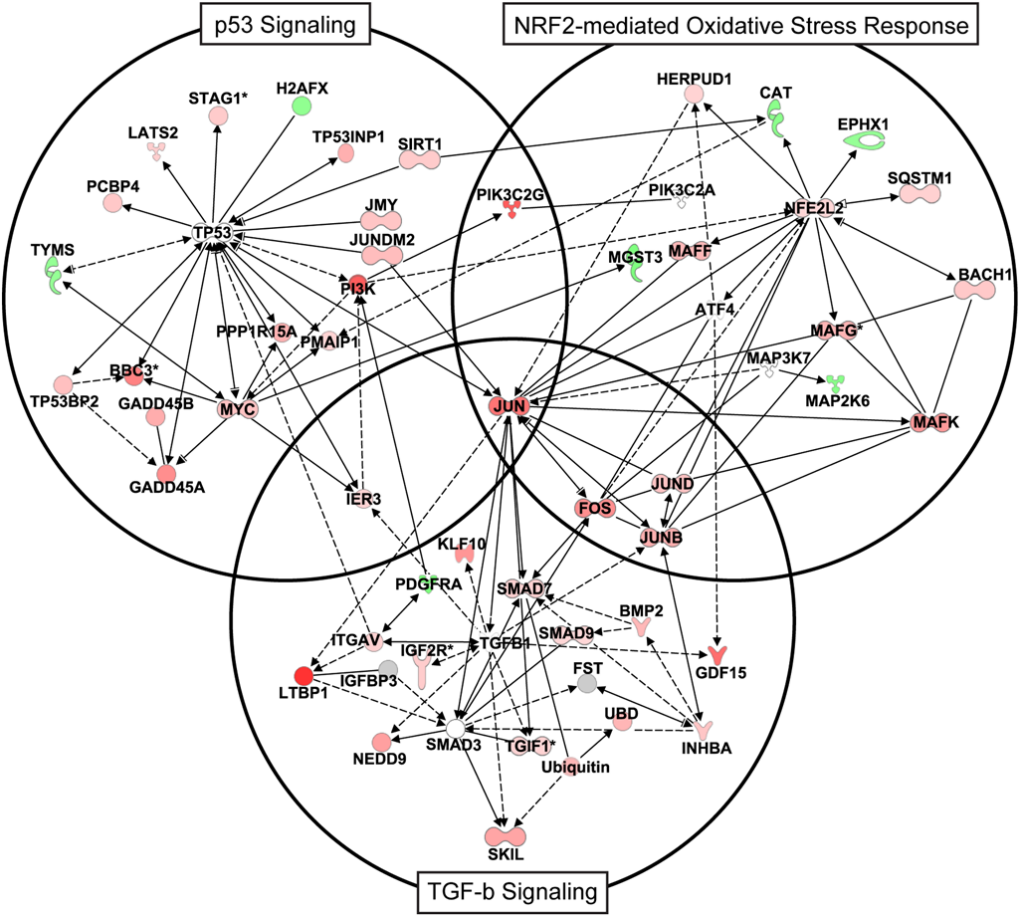
Ingenuity network diagram depicting interactions between the components of the p53, TGF-β1, and NFR2-mediated oxidative stress signaling pathways. Lines indicate known interactions between proteins (sold lines depicts direct and dashed lines depict indirect interaction). Gene expression data from 72 hrs post-infection was overlaid onto the network with genes showing increased and decreased expression (relative to mock) shown in red and green, respectively. For visualization, network was mainly restricted to genes showing regulation during infection and not all interactions between genes from different pathways are indicated.

TGF-β1 is the most likely candidate for exerting effects on hepatocytes that are consistent with the gene expression data indicating cell arrest and apoptosis. It is a potent inhibitor of cell growth of many cell types, including hepatocytes, and growth arrest occurs by blocking the cell cycle at middle and late G1 phase of cell cycle [13]. Although TGF-β1 itself was not induced, there was increased abundance of a significant number of genes associated with TGF-β1 signaling, particularly at 72 hrs post-infection. Many of these genes are either associated with the TGF-β1 signaling pathway (BMP2, TGIF1, SMAD7 and 9, MRAS, FOS, ROR1, PDGRFA, JUN, INHBA) and/or are known to be regulated by TGF-β1. The increased expression of Smad7, which provides a TGF-β1-induced negative feedback loop by inhibiting nuclear translocation of SMAD proteins, suggests that TGF-β1 is produced and interacting with receptors present on hepatocytes [14]. Interestingly, KLF10 (also known as TIEG) is a gene induced by TGF-β1 that induces the generation of ROS and the loss of mitochondrial membrane potential prior to death. This, together with the fact that p53 plays a key role in TGF-β1-induced growth arrest [15], may provide an important link between the three most significant pathways affected by HCV replication: TGF-β1 signaling, p53 signaling and the NFR2-mediated oxidative stress response (Figure 6). As indicated by the network analysis, there is extensive interaction between genes associated with these pathways.

### Common Regulation of Cell-Death– and Cytokine-Signaling–Related Genes during *In Vitro* Infection and Recurrent HCV Infection in Liver Transplant Patients

**Figure 7:**
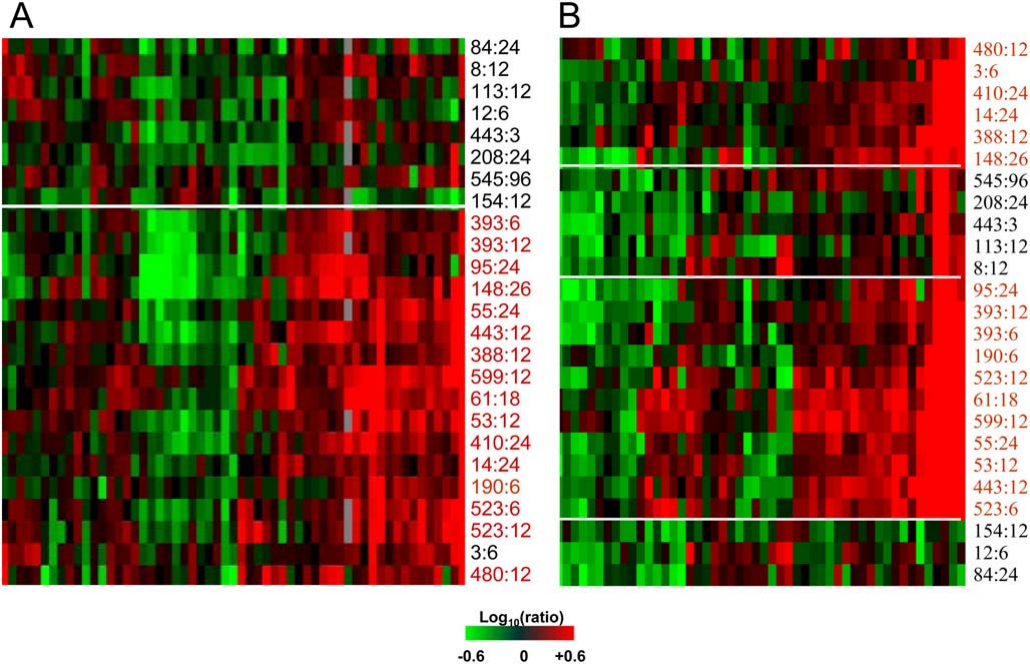
Common regulation of cell-death– and cytokine-related genes HCV-infected cultured hepatocytes and patient liver tissue. (A) Expression of cell-death–related genes in individual liver biopsies from HCV-infected liver transplant patients (n = 25). Two-dimensional hierarchical clustering was done using Resolver System software with an agglomerative algorithm, complete link heuristic criteria, and Euclidean correlation metric. Each column represents gene expression data from an individual experiment comparing a single HCV-infected liver tissue to a pool of normal, uninfected liver tissue. Heatmap depicts a subset of 96 genes from Figure S1 that are regulated (2-fold, p value<0.05) in at least 3 experiments. Patients with fibrosis are indicated in red text while non-fibrotic patients are shown in black text. (B) Expression of cytokine- related genes in liver biopsy tissue from HCV-infected liver transplant patients. Heatmap depicts a subset of 50 genes from Figure 5 that are regulated (2-fold, p value<0.05) in at least 3 experiments. Patients with fibrosis are indicated in red text while non-fibrotic patients are shown in black text.

As part of a separate study examining the progression of fibrosis in liver transplant patients with re-current HCV, microarray experiments were performed comparing individual patient liver biopsy tissue (n = 25) to a pool of normal, uninfected liver tissue. To determine the clinical relevance of transcriptional changes observed in HCV-infected Huh-7.5 cells, the expression of the cell death-related genes regulated at 72 hrs post-infection was assessed in liver tissue from these HCV-infected patients. As shown in Figure 7A, many of the genes that were induced during HCV infection in cell culture were also regulated during re-current HCV infection in liver transplant patients. Interestingly, the increased expression of a subset of these genes appeared to be associated with liver disease progression as they were, in general, more highly induced in patients who developed rapidly progressive fibrosis post-transplant (indicated in red text) than in patients who did not (black text). The fact that only a subset of the cell death genes regulated *in vitro* were regulated in liver tissue can likely be attributed to the much lower incidence of hepatocyte apoptosis and multiple cell types in the livers of chronically infected patients. A similar scenario was observed when the expression of cytokine signaling genes differentially regulated during HCV infection of Huh-7.5 cells was assessed in the patient cohort. A subset of these genes was more highly expressed in patients who develop recurrent liver disease (indicated in red text in Figure 7B). Significantly, some were identified by ANOVA (comparing patients with and without re-current disease post-transplant) as being statistically (P value<0.05) associated with fibrosis development. Collectively, these data demonstrate that, despite JFH-1 being somewhat of an atypical HCV, transcriptional changes which occur in HCV-infected Huh-7.5 cells parallel those which occur specifically during fibrosis development in HCV-infected patients.

## Discussion

This study represents the first report of global transcriptional profiling of HCV-J6/JFH-infected cultured human hepatoma cells. It is unique in that it examines the host response to *in vitro* infection during the early acute phase of infection. The host transcriptional response corresponds closely to the levels of HCV replication, with the most gene expression changes coinciding with peak intracellular viral load (72 hours). No changes in gene expression were observed in cells treated with UV-inactivated HCVcc, indicating that viral attachment/entry does not significantly impact host gene expression. It also indicates that the changes observed in the HCV-infected cells are dependent on HCV replication and not the interaction of secreted cellular factors present in the inoculum. In contrast, both replication-dependent and -independent transcriptional changes are observed during acute influenza virus infection [16]. This discrepancy may be a reflection of inherent differences in host response to acute versus chronic viruses. Chronic viruses such as HCV may have evolved to cause minimal impact upon entry into cells in an effort to delay cellular changes that may trigger an immune response, as evidenced by the minimal effect on host gene expression even at 24 hrs post-HCV infection. Similar results were obtained in transcriptional profiling of acute HBV infection in chimpanzees, where viral entry and expansion occurred in the absence of host gene regulation [17].

Significantly, the results of this study indicate that HCV has the potential to mediate direct cytopathic effects, suggesting that not all liver injury during chronic HCV infection is immune-mediated. The initial appearance of cytopathic effect, activated caspase-3 and the highest induction of cell death-related genes all coincided with peak viral loads, suggesting that intrahepatic HCV RNA levels play a role in hepatocyte cell death. While the role of viral load in HCV-liver disease remains controversial, there is evidence to suggest that higher replication rates are associated with more severe liver disease, particularly in the liver transplant setting. Significantly higher pre- and post-transplant serum HCV levels has been associated with cholestatic fibrosis, a severe form of hepatitis [18]. Similarly, elevated serum HCV pre-transplant is associated with accelerated HCV-induced allograft injury [19]. High levels of intrahepatic HCV in biopsies taken at early times post-transplant was found to be an independent predictor of progression to chronic active hepatitis [20]. In the non-transplant setting, *in situ* hybridization demonstrated an association between the number of hepatocytes harboring replicating HCV and severity of fibrosis [21]. Collectively, these studies suggest that elevated viral replication may cause increased liver injury.

Differences in viral load may actually provide an explanation for the discrepancy in the level of hepatocyte cell death that occurred *in vitro*, which involved the majority of cells, and during chronic HCV infection. The lack of important dsRNA signaling molecules in the hepatoma cell line Huh-7.5 used for this study allows higher levels of HCV replication than what is typically observed in the liver of chronic HCV patients (Walters, unpublished data), presumably due to decreased activation of an intracellular innate antiviral response. The balance of IFN-mediated suppression of HCV replication and HCV-mediated regulation of host innate antiviral pathways likely varies from cell to cell in infected livers. If the balance shifts more toward HCV control over innate antiviral signaling, then HCV levels within that cell may increase to a level that is incompatible with cell survival. HCV infection in Huh-7.5 cells likely reflects the extreme of this situation. Indeed, levels of HCV replication in Huh-7 cells are much lower than in Huh-7.5 cells and is associated with a delay in cell death. It is possible that apoptotic hepatocytes in infected livers also have higher levels of HCV replication than non-apoptotic cells, although this would be technically challenging to demonstrate.

A significant proportion of differentially expressed genes during *in vitro* HCV infection were associated with cell cycle checkpoint/arrest and subsequent induction of apoptosis, which may have been in response to oxidative stress/DNA damage. Also, the observed delayed growth kinetics of HCV-infected cells and flow cytometry analysis demonstrating fewer cells in S-phase, suggests that delayed cell cycle progression may be involved in HCV-mediated cytotoxicity. This is particularly interesting in light of recent studies where immuno-histochemistry of patient liver biopsies demonstrated that few hepatocytes which have entered the cell cycle go beyond G1 phase during chronic HCV infection [22],[23]. The G1 arrest observed in patient livers was associated with increased expression of p21, a cdk-cyclin inhibitor which causes G1 arrest after DNA damage. A correlation was observed between p21 expression and fibrosis severity, suggesting a link between delayed cell cycle progression and liver injury. Such results suggest the delay in cell cycle progression observed in HCV-infected Huh-7.5 cells is physiologically relevant. Consistent with these *in vivo* studies, many of the genes associated with arrest during *in vitro* HCV infection are involved in G1 arrest or transition from G1 to S phase. Interestingly, a link between perturbations in cell cycle control and pathogenesis has been observed in SIV infection in non-human primates, another chronic viral infection that can have different disease outcomes. Perturbation of the cell cycle within CD4 (+) T lymphocytes is characteristic of pathogenic HIV/SIV infection [24],[25],[26] and, similar to what is proposed in the current study, increased T lymphocyte susceptibility to apoptosis correlates with cell cycle perturbation [26]. Unlike the AIDS field, there is no animal model of HCV-associated liver disease in which to validate the biological significance of events which occur in cell culture models. To circumvent this challenge, gene expression data from *in vitro* HCV infection was integrated with an extensive database of patient liver microarray experiments. The intriguing finding that a higher induction of a subset of these genes was observed in HCV-infected patients with rapidly progressive fibrosis post-transplant, but not those HCV-infected patients lacking histological evidence of fibrosis, also suggests a potential role of cell cycle perturbations in HCV pathogenesis. However, it is important to note that this patient cohort is immuno-compromised and so it is uncertain if HCV-associated CPE causes significant liver injury in immuno-competent individuals.

Both gene expression profiling and flow cytometry analysis suggest that HCV-mediated apoptosis of Huh-7.5 cells is linked to perturbations in cell cycle progression. However, it is difficult to determine from the gene expression data if the cell cycle arrest is directly linked to apoptosis or if there other factors that are driving the arrested cells to undergo apoptosis. It is also not clear what factors are responsible for inducing the delay in cell cycle progression. Perturbation of the cell cycle may be mediated directly by HCV proteins, as has been observed in other viral infections including HIV (vpr) and HBV (x protein) [27],[28]. Due to the association between chronic HCV infection and development of hepatocellular carcinoma, there has been keen interest in the impact of HCV on cell cycle regulation. Core protein in particular has been implicated in impairment of G1 to S phase transition through multiple mechanisms, including induction of p21 expression and concomitant decrease in cdk2 activity [29], direct interaction and suppression of CAK activity [30], and stabilization of cell cycle inhibitor p27 [31]. Delayed progression through S-phase has been shown to be mediated both by NS2-mediated down-regulation of cyclin A [32] and NS5B induction of IFN-B [33]. NS5A-induced chromosome instability has been linked to aberrant mitotic regulation, including impaired mitotic exit [34]. It is unclear whether delaying cell cycle progression would be beneficial or detrimental to HCV replication. The liver is normally a quiescent organ and so hepatotropic viruses which establish chronic infections, such as HBV and HCV, may have evolved to replicate efficiently under such non-proliferating conditions. In support of this, production of infectious HCV in cultured hepatoma cells does not seem to be significantly impacted during growth arrest induced by either serum starvation or DMSO [35],[36].

The differential regulation of numerous genes associated with DNA damage/oxidative stress response, including many associated with the NRF2-oxidative stress response, suggests this as a possible mechanism of arrest. Oxidative stress has long been thought to play a key role in HCV pathogenesis and a potential link between HCV-associated perturbation of lipid metabolism genes and oxidative stress was observed in the SCID-Alb/uPA mouse model [5]. Specifically, HCV-infected animals showing induction of genes functioning in cholesterol biosynthesis, peroxisome proliferation, and β-oxidation also showed induction of genes which function in antioxidant cell defense, presumably due to generation of reactive oxygen species (ROS) during β-oxidation of fatty acids. In the current study, oxidative stress appears to be independent of lipid metabolism. No significant regulation of genes associated with cholesterol synthesis or enzymes involved in β-oxidation was observed at any time-point of infection. It is possible that the presence of viral gene products or replication directly results in the generation of ROS. Core protein has been implicated in perturbations in mitochondrial function, including release of cytochrome c and loss of membrane potential, as well as production of ROS in a variety of systems [37],[38],[39]. Consistent with the current study, expression of full-length HCV open reading frame was found to cause marked growth inhibition and increased intracellular ROS [40]. Preliminary global quantitative proteomic data showed multiple perturbations in the host proteome indicative of HCV-associated metabolic stress and ROS generation as early as 24 h post infection (Diamond *et al*, manuscript in preparation). Consistent with this idea, these perturbations were accompanied by a concomitant increase in proteins functioning in antioxidant cell defense.

Another possible mechanism of cell arrest could be through activation of TGF-β1 signaling. TGF-β1 is a potent inhibitor of cell growth and apoptosis of many cell types, including hepatocytes, and growth arrest occurs by blocking cell cycle at mid and late G1 phase [13],[14]. This is consistent with the gene expression data showing regulation of numerous genes involved in G1 arrest. Significant correlations between TGF-β1 polymorphisms, intensity of hepatocyte-specific TGF-β1 staining, serum TGF-β1 levels and degree of fibrosis have consistently been demonstrated in HCV-infected patients [41],[42],[43]. However, its role in hepatocyte apoptosis during HCV infection remains unclear. The possibility that TFG-β1 may be directly involved in HCV-associated cell cycle arrest is intriguing. While increased expression of TGF-β1 was not observed in the current study, the expression of numerous genes associated with TGF-β1 signaling pathway, and those known to be induced by TGF-β1, was elevated during infection. It is possible that very low levels of the cytokine are needed to activate the pathway, a scenario similar to what is observed with Type 1 IFN and ISGs. Furthermore, in an infected liver other cell types, including hepatic stellate cells, are an important source of TGF-β1. Chronic HCV infection is universally associated with the induction of ISGs in the liver but usually in the absence of detectable increased expression of IFN-α/β [5],[44]. TGF-β1 has been proposed as a potential therapeutic target for treatment of viral hepatitis and so a clear understanding of the role it plays in HCV pathogenesis is crucial [45]. Further study is warranted to determine if TGF-β1 is inducing cell arrest and also whether this is directly linked to apoptosis or simply sensitizing cells to apoptosis through alternative mechanisms, including the effects of oxidative stress.

Alternate mechanisms of HCV-induced cytopathic effects, such as through induction of ER stress, have been proposed [46]. However, there was little evidence for regulation of genes associated with either ER stress or the unfolded protein response in the current study. This is consistent with a recent study demonstrating that HCV-JFH-1 mediates apoptosis through a mitochondrion-mediated, caspase-3 dependent pathway in the absence of ER stress [47]. Although ER stress is associated with transcriptional regulation of a subset of genes, it is unclear if gene expression profiling would accurately detect ER stress and so it should not be ruled out as a possible contributor of apoptosis in the current study. Apoptosis has also been found to be mediated through the induction of the death ligand, TRAIL, and its receptors [48]. No increase in the expression of TRAIL, or its receptors, was observed in the current study, making it unlikely to be involved in apoptosis of Huh-7.5 cells. This is possibly related to the absence of functional RIG-I and TLR3 in these cells, which are key components of dsRNA signaling pathways. It is important to note that these alternative mechanisms of HCV-mediated cell death are not necessarily mutually exclusive, and multiple mechanisms of cytotoxicity may be involved in liver injury during chronic HCV infection.

Collectively, the gene expression data and flow cytometry analysis suggests that HCV infection is associated with perturbation of the cell cycle which may sensitize cells to apoptosis. Significantly, the integration of the *in vitro* and patient liver gene expression data also suggests that this process contributes to liver disease progression. These results suggest that despite the fact that HCV typically establishes persistent infections, events which occur during the very acute phase of infection of individual hepatocytes can determine the ultimate fate of the cell. During the time this manuscript was in preparation, a report was published describing altered expression of cell cycle and apoptotic proteins, as demonstrated using immunohistochemistry, in liver biopsies from chronic HCV patients [49]. However, the delay in cell cycle progression and apoptosis were observed in separate cell types (hepatocytes versus sinusoidal cells, respectively). The results of the current study demonstrate that cell cycle perturbation and apoptosis occur in the same cell, suggesting a direct link, and also provide additional insight into potential mechanisms of cell cycle perturbation, including oxidative stress and TGF-β1 signaling. Further study is warranted to more clearly define the mechanism of HCV-associated cell cycle perturbation. This may provide significant insight into the pathogenesis of HCV infection with the possibility of identifying novel therapeutic targets.

## Materials and Methods

### Generation of HCVcc Stocks and Experimental Infections

Huh 7.5 cells (human hepatoma) were electroporated with 1 µg of *in vitro* transcribed RNA from the chimeric HCV genome J6/JFH (see reference 7); five identical electroporations were performed. Cells were expanded three times following electroporation and supernatants were pooled and used to infect naïve Huh 7.5 cells. Following a single expansion of infected cells, virus containing supernatants were collected, pooled, and used to again infect naïve Huh 7.5 cells. This process was repeated a total of five times and resulted in the generation of a relatively high titer (2×10^5^ TCID_50_/ml) and large volume (∼500 mls) of stock virus (HCVcc). Approximately one-half of the HCVcc stock was exposed to UV light for 60 seconds, using a Stratalinker UV light box, and served as a non-infectious control (UV-HCV). In addition, during the multiple passages of HCVcc on naïve cells, a mock control sample was generated by passing conditioned media along in parallel. For infections, Huh 7.5 cells were seeded at a density of 3×10^6^ cells/plate on p150 plates and treated for ∼8 hours with 20 mls of supernatant containing virus (HCVcc), UV-inactivated virus (UV-HCV), or conditioned media (mock). This amounted to a moi of ∼1.3. Following initial infection, the supernatant was replaced with fresh media and incubated until harvest at 24, 48, 72, 96, and 120 hours post-infection.

### Isolation of Total Cellular RNA

Following removal of supernatant, cells were washed once with PBS and then scraped from the plate in ice cold PBS. RNA was isolated from approximately 10^6^ cells using RNeasy mini prep kit with an on-column DNase treatment, following the manufacturer protocol (Qiagen).

### Immuno-Fluorescence Staining

Cells were fixed in −20°C methanol or 1% paraformaldehyde (in PBS at room temperature) for 15–20 minutes. Following a series of PBS rinses, the cells were blocked in 1% BSA/0.2% skim milk in PBS for 30–60 minutes at room temperature. Cells were incubated in primary antibody (diluted in 0.5% Tween-20 in PBS) overnight at 4°C; mouse anti-NS5A (1∶2000, Clone 9E10, ref. 21) and rabbit anti-activated caspase-3 (1∶500, Cell Signaling). Click-iT EdU chemistry was performed following manufacturer protocol (Invitrogen); 10 µM EdU labeling for 3 hours and 30 minute reaction with AlexaFluor 488 azide (1∶400 dilution). Secondary antibodies are AlexaFluor conjugated and used at 1∶1000 dilution; goat anti-mouse AlexaFluor488 and goat anti-rabbit AlexaFluor594.

### Flow Cytometry Analysis

Cells were trypsinized, washed twice with ice cold PBS and fixed 1% paraformaldehyde for 20 minutes. Following a series of PBS rinses, the cells were blocked and permeabilized in 0.1% FBS/0.1% saponin in PBS. HCV infected cells were detected using an anti-NS5A antibody (clone 9E10) directly conjugated with the AlexaFluor647 fluorophore (according to manufacturer protocol; Invitrogen, A20173). Proliferating cells were detected using Click-iT EdU chemistry, as described above, following manufacturer guidelines for flow cytometry analysis (Invitrogen); AlexaFluor 488 azide (1∶400 dilution). Flow cytometry was performed on a BD FACSCalibur machine with analysis done using FlowJo software (version 8.7.1).

### Human Liver Tissue Samples

Core needle liver biopsies were collected from patients at the University of Washington All patients gave informed consent to protocols approved by the Human Subjects Review Committee at the University of Washington. Normal, uninfected liver tissue (n = 10) was obtained from donor livers that were considered unacceptable for liver transplantation. These uninfected samples were pooled to create a standard normal liver reference that was used for all microarray experiments using patient tissue. Fibrosis was graded by a single liver pathologist using the Batts-Ludwig grading system [50].

### Expression Microarray Format and Data Analysis

Microarray format, protocols for probe labeling, and array hybridization are described at http://expression.microslu.washington.edu. Briefly, a single experiment comparing two mRNA samples was done with four replicate Human 1A (V2) 22K oligonucleotide expression arrays (Agilent Technologies) using the dye label reverse technique. This allows for the calculation of mean ratios between expression levels of each gene in the analyzed sample pair, standard deviation and P values for each experiment. Spot quantitation, normalization and application of a platform-specific error model was performed using Agilent's Feature Extractor software and all data was then entered into a custom-designed database, Expression Array Manager, and then uploaded into Rosetta Resolver System 7.0 (Rosetta Biosoftware, Kirkland, WA) and Spotfire Decision Suite 8.1 (Spotfire, Somerville, MA). Data normalization and the Resolver Error Model are described on the website http://expression.viromics.washington.edu. This website is also used to publish all primary data in accordance with the proposed MIAME standards [51]. Selection of genes for data analysis was based on a greater than 95% probability of being differentially expressed (*P*≤0.05) and a fold change of 2 or greater. The resultant false positive discovery rate was estimated to be less than 0.1% (Walters, unpublished data). Ingenuity Pathway Analysis (IPA) software and Entrez Gene (www.ncbi.nlm.nih.gov/sites) were used for gene ontology analysis.

### Quantitative RT-PCR

Quantitative real-time PCR (RT-PCR) was used to validate the gene expression changes and measure intrahepatic HCV RNA. Total RNA samples were treated with DNA-free DNase Treatment and Removal Reagents (Ambion, Austin, TX). Reverse transcription was performed using random hexamer primers and Taqman RT reagents (Applied Biosystems, Foster City, CA). Real-time PCR was performed using an ABI 7500 Real Time PCR system and Taqman chemistry. Each target was run in quadruplicate with Taqman 2× PCR Universal Master Mix and a 20 µL total reaction volume. Primer and probe sets for relative quantification were selected from the Assays-on-Demand product list (Applied Biosystems) including two endogenous controls, GAPDH and 18 S ribosomal RNA. Quantification of each gene, relative to the calibrator, was calculated by the instrument, using the equation 2^−ΔΔCT^ within the Applied Biosystems Sequence Detections Software version 1.3. Probes used for analysis (Applied Biosystems): Human genes: eukaryotic 18S rRNA (Catalogue No. Hs99999901_s1); ATF3 (catalogue No. Hs00910173_ml), MKi67 (catalogue No. Hs01032443_ml), MCM4 (catalogue No. Hs00381539_ml), MCM6 (catalogue No. Hs00195504_ml), TGIF1 (catalogue No. Hs00545014_ml), CAT (catalogue No. Hs00156308_ml), SMAD7 (catalogue No. Hs00998193_ml), GADD45A (catalogue No. Hs00169255_ml), GADD45B (catalogue No. Hs00169587_ml)

Primer and probe sets for absolute quantification of intrahepatic viral load were designed based on sequences of HCV 1a armored RNA (Ambion Diagnostics, Austin, TX) using Primer Express (version 3). A standard curve was made from six serial dilutions of HCV 1a armored RNA (Ambion Diagnostics) with a known viral copy number. The PCR efficiency was determined by the slope of the standard curve Standard curve analysis and viral load was determined using the Applied Biosystems SDS Software 1.3 (Applied Biosystems, CA). Total RNA was DNase treated prior to cDNA synthesis via reverse transcription and all samples were processed with equal mass amounts of total RNA [52]. All measurements were taken in quadruplicate with negative and non-template controls. Primer and probe sets consisted of F: CAC TCC CCT GTG AGG AAC TAC TG, R: GCT GCA CGA CAC TCA TAC TAA CG, and P: 6FAM-TTC ACG CAG AAA GC-MGBNFQ and were designed from the 5′UTR using Primer Express 3.0 (Applied Biosystems, CA). Quantification of HCV RNA levels was performed on the same total RNA sample that was used for the microarray experiments.

## Supporting Information

Figure S1 Expression profiles of cell death-associated genes in HCV-infected Huh-7.5 cells. Heatmap depicts 118 genes regulated (2-fold, p value<0.05) in at least 1 experiment. Each column represents gene expression data from an individual experiment comparing either UV-inactivated HCV or HCV -treated cells relative to time-matched mock-treated Huh-7.5 cells. Genes shown in red were up-regulated, genes shown in green down-regulated and genes in black indicate no change in expression in HCV-infected cells relative to uninfected cells.
(2.36 MB TIF)

Table S1 Primary sequence name and fold-change of genes differentially regulated during HCV infection.
(0.39 MB XLS)
