# Supplementary figures and images for: Genomic Analysis Reveals a Potential Role for Cell Cycle Perturbation in HCV-Mediated Apoptosis of Cultured Hepatocytes
Source: PLoS Pathog. 2009 Jan 16;5(1):e1000269. doi: 10.1371/journal.ppat.1000269 (PMC2613535; doi:10.1371/journal.ppat.1000269)

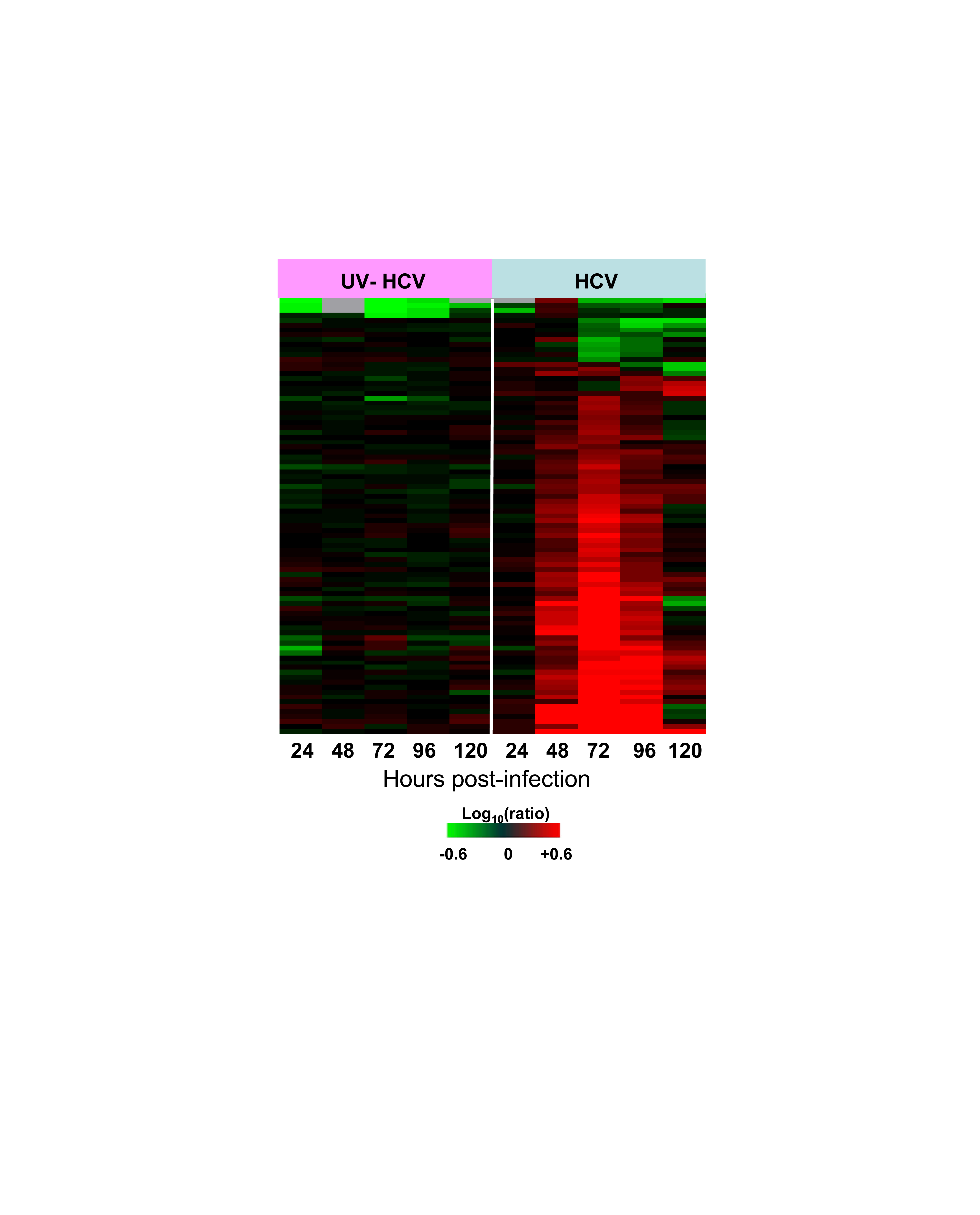

Supplement: Figure S1 — Expression profiles of cell death-associated genes in HCV-infected Huh-7.5 cells. Heatmap depicts 118 genes regulated (2-fold, p value<0.05) in at least 1 experiment. Each column represents gene expression data from an individual experiment comparing either UV-inactivated HCV or HCV -treated cells relative to time-matched mock-treated Huh-7.5 cells. Genes shown in red were up-regulated, genes shown in green down-regulated and genes in black indicate no change in expression in HCV-infected cells relative to uninfected cells. (2.36 MB TIF) [file ppat.1000269.s001.tif]
